# Development of an Obesity Management Ontology Based on the Nursing Process for the Mobile-Device Domain

**DOI:** 10.2196/jmir.2512

**Published:** 2013-06-28

**Authors:** Hyun-Young Kim, Hyeoun-Ae Park, Yul Ha Min, Eunjoo Jeon

**Affiliations:** ^1^College of NursingEulji UniversityDaejeonRepublic of Korea; ^2^College of NursingSeoul National UniversitySeoulRepublic of Korea; ^3^Research Institute of Nursing ScienceSeoul National UniversitySeoulRepublic of Korea

**Keywords:** obesity management, ontology, nursing process

## Abstract

**Background:**

Lifestyle modification is the most important factor in the management of obesity. It is therefore essential to enhance client participation in voluntary and continuous weight control.

**Objective:**

The aim of this study was to develop an obesity management ontology for application in the mobile-device domain. We considered the concepts of client participation in behavioral modification for obesity management and focused on minimizing the amount of information exchange between the application and the database when providing tailored interventions.

**Methods:**

An obesity management ontology was developed in seven phases: (1) defining the scope of obesity management, (2) selecting a foundational ontology, (3) extracting the concepts, (4) assigning relationships between these concepts, (5) evaluating representative layers of ontology content, (6) representing the ontology formally with Protégé, and (7) developing a prototype application for obesity management.

**Results:**

Behavioral interventions, dietary advice, and physical activity were proposed as obesity management strategies. The nursing process was selected as a foundation of ontology, representing the obesity management process. We extracted 127 concepts, which included assessment data (eg, sex, body mass index, and waist circumference), inferred data to represent nursing diagnoses and evaluations (eg, degree of and reason for obesity, and success or failure of lifestyle modifications), and implementation (eg, education and advice). The relationship linking concepts were “part of”, “instance of”, “derives of”, “derives into”, “has plan”, “followed by”, and “has intention”. The concepts and relationships were formally represented using Protégé. The evaluation score of the obesity management ontology was 4.5 out of 5. An Android-based obesity management application comprising both agent and client parts was developed.

**Conclusions:**

We have developed an ontology for representing obesity management with the nursing process as a foundation of ontology.

## Introduction

### Overview

Advancements in socioeconomic status and lifestyle changes in Korea have led to an increasing number of obese people and a consequent increase in the importance of obesity management to prevent illness and promote health among the population [1]. There has been an emphasis on the development and implementation of various programs to help clients who wish to manage obesity effectively. Since lifestyle modification is the most important aspect of obesity management, such programs must be able to enhance the client’s participation in voluntary and continuous weight control [2-4].

This study investigated two strategies for empowering clients to effectively manage their obesity: (1) use of mobile devices, which are now an integral part of everyday life in many countries [5], and (2) use of evidence-based knowledge. It is important to provide a nursing service whenever a client requires it, without time or space limitations, and especially for a problem such as obesity that requires continuous self-monitoring. Previous research has demonstrated that mobile devices such as smartphones are very effective tools for self-monitoring in obesity management [6,7]. In addition, mobile devices allow users to input and communicate data in real time, regardless of their physical location [8].

Some clients lack knowledge regarding how to manage obesity, and the information provided through mobile devices should be based on the available evidence. The best sources for evidence-based knowledge are published clinical practice guidelines. The content for obesity management should vary with the health behavior and diet patterns of the individual client. The effectiveness of obesity management can be improved if time-stamped behavioral data are collected and tailored coaching is provided remotely, based on published clinical practice guidelines, using mobile devices [2,9].

Since these guidelines are written in natural language, it is necessary to translate them into a computer-interpretable format. Ontology is a computer-interpretable knowledge model for formalizing and representing shared concepts in a specific domain of interest. It is considered to be a highly effective way of improving the integration, interoperability, and sharing of data. Moreover, the enhanced reusability of clinical data and evidence-based knowledge support clinical decision making by explicitly defining and delivering semantic concepts in a specific domain [10-12].

### Background

**Figure 1:**
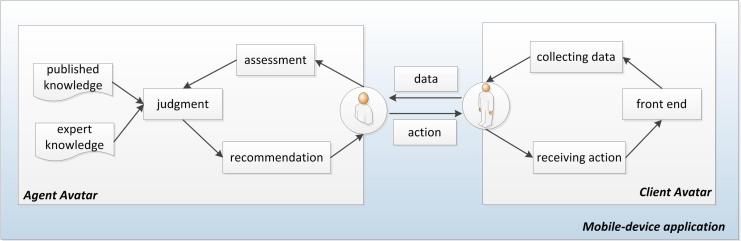
The Health Avatar Project model.

This project is a part of the Health Avatar Project in Korea, which promotes health and manages health problems using personal data and knowledge in virtual space. The health avatar consists of an agent avatar, which is a representation of the expert knowledge or published knowledge (such as clinical practice guidelines), and a client avatar, which is a representation of personal data from molecular to community levels (Figure 1). The health avatar collects data from the client avatar and provides it with tailored care solutions based on the personal data and knowledge or evidence provided by the agent avatar. The client avatar collects and sends personal data to the agent avatar. Upon receiving that personal data, the agent avatar makes judgments and provides the client avatar with tailored recommendations.

The obesity management application is a type of health avatar. The client avatar can be represented by data such as weight, height, abdominal circumference, physical activity, and diet, while the agent avatar can be represented by the clinical practice guidelines published by the National Institute for Health and Clinical Excellence (NICE). Personal data such as weight and height, and tailored recommendations, alarms, or reminders such as “educate about low-calorie diet” are transferred between the client and agent avatars. The front end serves as a user interface to collect these personal data and display tailored recommendations suggested by the agent avatar.

### Objectives

This study considered two objectives during the development of the obesity management ontology. The first objective was to identify concepts related to client participation in behavioral modification for obesity management, since it is important to identify the factors that motivate and encourage participation in the process. The possibility of using the nursing process as a foundation of ontology was explored, since that process is a patient-focused clinical reasoning method for nursing problems, and each phase of the nursing process emphasizes the active participation of the client [13]. The second objective was to minimize information exchange between the application and the database when providing tailored interventions. The obesity management application program developed in this study operates on a platform that requires real-time, two-way data communication with the database, storing an enormous volume of individual life-log data. In addition, this application program could ultimately share the database and platform with other health care management services, such as those for diabetes and dyslipidemia. Thus, it is important to distinguish between the types of data used in different phases of the service to minimize data communication between the database and the application.

With this background, a clinical practice guideline-based obesity management ontology was developed by identifying the concepts and relationship between concepts according to the nursing process, using ontology development methods that are used in the biomedical arena. In addition, concepts describing client participation in the application program, which is the most important aspect of obesity management, were identified and reflected in the ontology, and finally, the ontology was evaluated.

## Methods

### Overview

An obesity management ontology was developed using the General Formal Ontology method [14], which comprises seven phases: (1) defining the scope of obesity management, (2) selecting a foundational ontology, (3) extracting the concepts, (4) assigning relationships among these concepts, (5) evaluating representative layers of ontology content, (6) representing the ontology formally using Protégé (an open-source ontology editor and knowledge-based framework), and (7) developing a prototype Android application for obesity management.

### Defining the Scope for Obesity Management

To determine the scope of obesity management services for adult clients, a guideline developed by NICE [15] was referenced as an evidence-based clinical practice guideline. This guideline was chosen because there is a version for patients/clients. Five experts (1 physician who cares for obese patients, 2 nurses who have participated in obesity-related research for over 2 years, and 2 informatics nurses with more than 5 years of work experience) participated in this process. Intervention strategies were selected from the guideline using a consensus method.

### Selecting a Foundational Ontology for Obesity Management

We reviewed top-level ontologies such as Basic Formal Ontology and a theoretical framework to determine the most appropriate foundational ontology for obesity management, thus minimizing errors during the creation of a domain-specific ontology [10,16,17]. The first criterion used for selecting a foundational ontology was whether it meets two of the purposes of an obesity management ontology: (1) enhancing client participation and (2) minimizing real-time data traffic when providing client-specific interventions. The second criterion used was whether it can be used as a basic framework when translating text-based guidelines into computer-interpretable knowledge [10,12]. An initial ontology was constructed according to the information types used during the care process for obesity management. This initial ontology was elaborated using concrete concepts extracted in the next phase.

### Extracting Concepts From the Guidelines on Obesity Management

The concepts needed to assess the client, make a diagnosis, determine client-specific interventions, and evaluate the outcome of those interventions within the scope of obesity management were extracted. These concepts included those used to represent not only personal information but also client-specific interventions from the guidelines based on personal data. This phase considered the concepts needed to enhance the client participation, together with ways to operationalize those concepts.

### Assigning Relationships Among These Concepts

The extracted concepts were specified as classes, individuals, and relationships between classes and individuals in order to define the obesity management ontology according to the foundational ontology determined in the second phase [18]. The “OBO relationship ontology of the Open Biological and Biomedical Ontologies” (OBO) [19,20] and the linkage concept hierarchy of SNOMED CT (Systematized Nomenclature of Medicine Clinical Terms) were referred to when defining the relationships.

### Evaluation of Representative Layers of Ontology Content for Obesity Management

The content of the obesity management ontology was evaluated using scenarios via peer-review by 3 ontology and domain experts [12,21,22]. This was a necessary modeling process to evaluate the quality and consistency of the ontology [23]. We evaluated whether the selected foundational ontology was sufficiently valid to represent the obesity management ontology. In addition, we determined whether the obesity management ontology developed with concepts extracted from clinical practice guideline was representative of the concepts identified from real clinical cases. Representation/semantic-layer evaluation criteria were used as one of the basic internal dimension evaluations. Nine-item Likert scales with 5-point scores (from “strongly agree” to “strongly disagree”) were used as evaluation tools. The nine items were as follows: match between formal and cognitive semantics, consistency (no formal contradictions), clarity (context and background knowledge), explicitness (understanding the concept semantics), interpretability (formal, informal, logical specification, and documentation), accuracy (fit between ontology and corpus terms), comprehensiveness (extent of the target domain covered), granularity (fine-grained coverage vs loose coverage), and relevance (for users) [22]. Three experts with experience in ontology development participated in the evaluation, with five case studies on obesity management published in Korean used as cases for evaluation [24,25].

### Formal Representation of the Ontology With Protégé

The extracted concepts and relationships were formalized using Protégé. The feasibility of the representation of the nursing-process ontology based on Protégé was assessed.

### Development of a Prototype Application for Obesity Management

A prototype application was developed based on an obesity management ontology incorporating the two key features of client participation and minimizing information exchange between the application and the database.

## Results

### The Scope for Obesity Management

The clinical practice guidelines on obesity reported by NICE (2006) [15] include lifestyle interventions, behavioral interventions, physical activity, dietary advice, pharmacological interventions, and surgical interventions as strategies for the management of overweight and obesity. In this study, lifestyle interventions, behavioral interventions, physical activity, and dietary advice were proposed as obesity management strategies. With respect to behavioral interventions, keeping a diary of physical activity and diet, and cognitive restructuring such as realistic goal setting were included according to the scope and sustainability of clients’ behavioral change. Pharmacological and surgical interventions were excluded because these strategies cannot be self-managed by clients, as they require the intervention of a health care provider.

### A Foundational Ontology: the Nursing Process

The nursing process met the requirements of promoting client participation and minimizing data transactions between databases and the obesity management application in this study. The nursing process comprises five cyclical phases: assessment, nursing diagnosis, outcome identification, implementation, and evaluation. The nursing process as a framework for nursing practice is a systematic, patient-centered, and goal-oriented method with the following phases: (1) identifying client status and need, (2) making nursing diagnoses based on the available client data, (3) determining the expected outcome for a nursing problem for the client, (4) determining and implementing the best nursing interventions to reach the goal, and (5) evaluating whether the expected outcome has been reached. This framework was selected as a foundation of ontology because it describes well both the obesity management process and the client participation therein. In obesity management, the expected outcome is determined by the clients, the evaluation is performed repeatedly to determine whether the outcome has been reached, and the tailored best intervention is implemented to reach expected outcomes based on the client’s assessment data.

**Figure 2:**
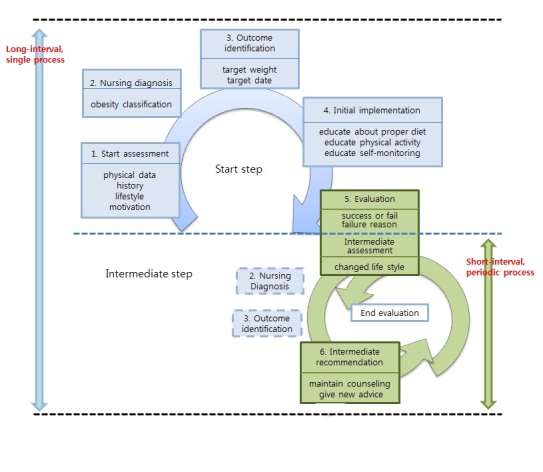
Cyclical process for obesity management.

Figure 2 illustrates the nursing process used for obesity management as a foundation of ontology. The nursing process can be subdivided into five phases according to the time taken to access the application when specifying the obesity management ontology. This was represented as a dynamic ontology with a spiral reflecting two cyclical nursing processes that are connected to each other. The blue color in the figure (ie, boxes 1, 2, 3, and 4) indicates the initial process of the obesity management service from the beginning to the end, while the green color (ie, boxes 5 and 6) indicates the repetitive process of obesity management from the beginning of the revisit to the service until a client reaches his or her identified outcome. These two cyclical nursing processes contribute to minimizing the amount of data transactions. The nursing diagnosis inferred using the personal data and evidence-based knowledge in a single, long-interval process can be reused in short-interval periodic processes; therefore, data needed in short-interval, periodic processes would be minimized.

**Figure 3:**
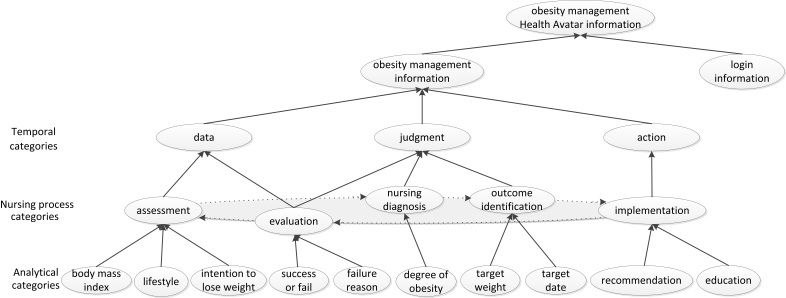
Initial obesity management ontology based on the nursing process.

Figure 3 shows the obesity management information, which in part reflects the cyclical characteristics of the service provision and login information, with client information guiding that cyclic process. This initial nursing process ontology was elaborated based on these information characteristics. The model has three levels of category: temporal, nursing process, and analytical. The temporal level was an abstract category corresponding to the obesity management. This was divided into assessment, nursing diagnosis, goal, outcome evaluation, and implementation phases, according to the nursing process. The analytical category shows the data linkage to each phase of the nursing process. In the assessment phase, data are collected from a client, and the nursing diagnosis phase defines the degree of obesity using the data collected in the assessment phase. A target weight loss and the date by which to achieve that goal are set in the goal phase, while the intervention for obesity management is provided in the implementation phase. Finally, the state of the client after the intervention is defined in the outcome evaluation phase by comparing the goal with the reassessment data.

### Concepts Extracted From the Guidelines on Obesity Management

The concepts required to make nursing diagnoses, generate client-specific interventions to enable the individual to reach the goal that they have set, and evaluate outcomes in the obesity management process were extracted. In total, 127 concepts were identified (Multimedia Appendix 1). The extracted concepts included data collected in the assessment, the degree of obesity, interventions, and outcomes categories. Examples include personal information such as sex, height, weight, waist circumference, intention to lose weight, and risk factors that the client entered when they accessed the application for the first time, and the calorie gap calculated using the diet and physical activity monitoring data. Other examples are a nursing diagnosis such as “obese due to lack of physical activity” made by the application using data entered by the client, and an intervention such as “educate about physical activity” recommended by the application based on the nursing diagnosis.

### Relationships Between Classes and Individuals and Relationships Based on These Concepts

The 127 concepts extracted in the previous phase were arranged as classes and individuals depending on the foundational ontology, using the relationship-linking classes listed in Table 1.

### Results of Evaluation of the Obesity Management Ontology

The evaluation scores of the nursing-process-based obesity management ontology for various criteria are presented in Table 2. All nine items scored above 4, with interpretability and consistency items scoring 5 points. The category with the lowest grade was the granularity criterion. Ontology and domain experts recommended that the concepts representing activity level and diet should be defined in more detail.

**Table 1:** Relationship-linking classes in the obesity management ontology.

Relationship name	Defined at concept
Part of	Relationship between class and subclasses
Instance of	Relationship between instances and class
Derives from	Relationships of nursing diagnosis and evaluation to assessment data
Derives into	Relationships of assessment data to nursing diagnosis and evaluation
Has plan	Relationships of nursing diagnosis and evaluation to implementation
Followed by	Relationships of implementation to nursing diagnosis and evaluation
Intention	Relationship of nursing diagnosis to goal identification

**Table 2:** Grades of evaluation on the representation layer.

Criterion	Score		
	Mean	Minimum	Maximum
Match between formal and cognitive semantics	4.7	4	5
Clarity	4.0	4	5
Explicitness	4.3	4	5
Interpretability	5.0	4	5
Accuracy	4.7	4	5
Consistency	5.0	5	5
Comprehensiveness	4.3	4	5
Granularity	4.1	4	4
Relevance	4.8	4	5
Total	4.5		

### Artifact Using Protégé

**Figure 4:**
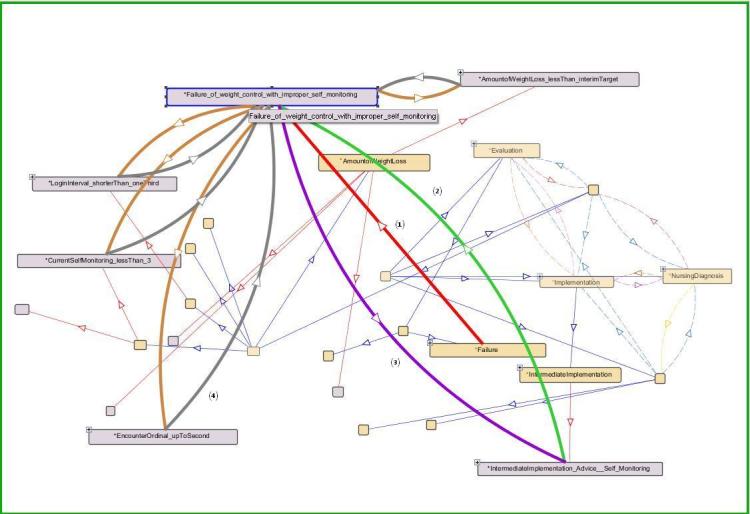
Some of the relationships among classes represented using Protégé.

Figure 4 shows some of the relationships among the classes represented by obesity management using Protégé. The rectangles represent classes and the arrows represent the relationship between the 127 concepts. The color of the arrow reflects the relationship among the classes and individuals. For example, red arrows (n=1) represent the relationship between each individual and class, which is “instance of”; green arrows (n=2) mean the relationship of “part of”; purple arrows (n=3) represent “followed by”, which is the relationship between implementation and evaluation in this figure; and gray arrows (n=4) represent “derives into”, which is the relationship between the assessment data and evaluation.

### A Prototype Application for Obesity Management

**Figure 5:**
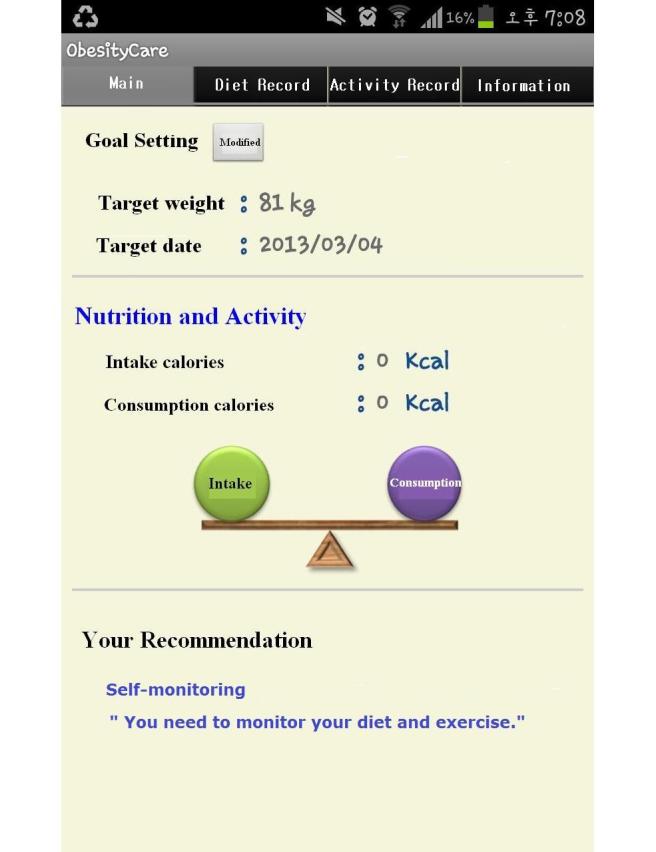
Screenshot showing an example of a client-specific recommendation.

The prototype application consisted of two parts: an agent avatar and a client avatar as described in the Figure 1. The agent avatar as a knowledge base was developed using an XML schema made from the obesity management ontology including 37 classes and 90 individuals in Protégé. The XML schema was translated into database schema with 11 tables for assessment, diagnosis, and outcome identification. In addition, classes such as diagnosis, implementation, and evaluation in XML were converted into Java classes with methods to call recommendations according to data entered, which were linked to database. The client avatar as a user interface consisted of 41 screens to collect client’s health data and deliver recommendations provided from the agent avatar. With JDK (Java development kit) 1.7.0, Eclipse having ADT (Android Development Tool) Plugin for Eclipse, and the Android SDK platform 4.0.3, the client avatar and the agent avatar were integrated. Figure 5 is a screen shot providing one of tailored recommendations.

## Discussion

### Principal Findings and Conclusions

Ontology is an effective way of representing specific domain knowledge in the field of biomedicine. It is also used for knowledge model development to support the clinical decision making of health care providers [18,26,27]. Most of the published ontologies have been developed to aid health care providers in clinical decision making [28,29]. However, the ontology developed in this study was designed to be used with a mobile-device-based application to provide tailored interventions to clients who want to manage their obesity by themselves.

The nursing process was used as a foundation of ontology to describe the client participation in obesity management. Since the nursing process is a scientific and systematic problem-solving framework that allows the client to participate in their own care [13], it was considered a valid representation of the client participation in obesity management. The findings of this study confirm that belief.

The client was considered to be the principal agent of goal setting and outcome evaluation in obesity management. The nursing-process–based obesity management ontology developed in this study allows the client to participate in setting a goal, such as a target weight loss and duration to reach to the target weight, and to monitor and evaluate their behavioral modification toward obesity management. To this end, a set of concepts was introduced to describe whether the goal and rate of weight loss were realistic and adequate, and another set was designed to describe whether the obesity management behavior was adequate based on the self-monitoring data.

One of the success factors in the existing Web-based intervention programs is login intervals by clients reflecting client participation [3]. The ontology developed in this study reflects this by introducing a concept to describe the continuity of the obesity management process based on the interval between two logins. If a client does not use the application within one-third of the target interval, which is the criterion used to judge the continuity of the care process, it was considered that the continuity of the obesity management process was deficient. In that case, the client must restart the long-interval, single process from the assessment point and will have to set a new goal. In order to promote the continuity of the obesity management process, reminder and alarm functions were implemented.

Another key characteristic of the nursing process is that it comprises cyclical phases. In this study, two cyclical nursing processes were connected to each other based on the sequences of logins to the application in our obesity management ontology: the first and second cycles of the nursing process correspond to the first visit and revisits, respectively. At the first visit, the degree of obesity is inferred based on height, weight, abdominal circumference, sex, and past history, and a goal is set. From the second visit, obesity management is evaluated by comparing current weight with the defined outcome. Based on the login information, which is used to guide the cyclical process of obesity management, if the client is either a first-time user of the application or a previous user who did not meet the continuity criteria, a long-interval, single process will start; otherwise, a short-interval, periodic process starts. This feature minimizes the real-time data traffic.

The obesity management ontology developed in this study was compatible with the Initial Clinical Information Ontology of the Danish General Electronic Patient Journal Conceptual Model [17]. This Danish model was introduced to clearly explain the clinical information created during the general care process using ontology. Even though the nursing process model used in this study as one of the care-process models was developed specifically to describe an obesity management process, it is compatible with the Danish model because the nursing process can be regarded as a type of general care process. However, some of the processes differ between the model developed in this study and the Danish model. The Danish model categorized the process into two different groups, ie, observation and intervention, whereas the model developed in this study included five different phases of the care process: assessment, nursing diagnosis, outcome identification, implementation, and evaluation.

Concepts extracted based on the nursing process are specified as classes and individuals. The relationships between these concepts were extracted from the OBO [19,20] and the linkage concept hierarchy of SNOMED CT. No new relationships were defined in this study. Thus, the ontology developed in the present study based on the nursing process has proved to be sufficiently universal to be integrated with others, since all of these concepts describing relationships are from the OBO and SNOMED CT linkage concept hierarchy. This is important because this study is a part of the Health Avatar Project. The design of this mobile-device-based ontology was based on three major criteria: practical relevance, incorporation of existing standards and ontologies, and extensibility towards future relevant domains [30].

The obesity management ontology developed herein was evaluated at a representation layer of the basic internal layers from various dimensions of the ontology [22]. The representation layer was evaluated for ontology semantics, with nine items. It was found that the concepts and the relationships between concepts used for obesity management were well represented, with a score of 4.5 out of 5. Furthermore, the nursing process was found to be adequate as a foundational ontology for obesity management ontology development. A nursing process with five distinctive phases with unique characteristics may have helped to improve the consistency of the ontology.

### Limitations

This study was subject to the following limitations. First, the developed ontology cannot be used for other types of nursing problems because the concepts used in its development were extracted specifically from obesity management guidelines. We recommend further development of this ontology for other types of nursing problems to determine whether the nursing process can be used as a foundation of ontology to represent nursing care in general. Second, the effectiveness of the prototype Android application developed in this study was not tested. The effectiveness of the application will be established in another study in the near future.

## Multimedia Appendix 1

Some of the concepts extracted from the guidelines.

## Abbreviations

NICE: National Institute for Health and Clinical Excellence
OBO: Open Biological and Biomedical Ontologies
SNOMED CT: Systematized Nomenclature of Medicine Clinical Terms
XML: Extensible Markup Language
