# Supplementary material for: Development of an Obesity Management Ontology Based on the Nursing Process for the Mobile-Device Domain
Source: J Med Internet Res. 2013 Jun 28;15(6):e130. doi: 10.2196/jmir.2512 (PMC3713932; doi:10.2196/jmir.2512)
Supplement: Supplementary file 1 [file jmir_v15i6e130_app1.pdf]

Multimedia Appendix 1. Table—Some of the concepts extracted from the guidelines.

Concepts			
**Login**			
	Contiguity		
		Yes	
		No	
**Assessment**			
	Initial assessment		
		Past body mass index	
			<25 kg/m^2
			≥25 kg/m^2
		Current body mass index	
			<25 kg/m^2
			≥25 kg/m^2 and <30 kg/m^2
			≥30 kg/m^2
		Intention to lose weight	
			Yes
			No
		Past average calorie intake gap	
			≥500 kcal
			<500 kcal
		Past average physical activity	
			More than three times per week and >30 minutes per week
			Less than three times per week and <30 minutes per week
	Intermediate assessment		
		Current average calorie intake gap	
			≥500 kcal
			<500 kcal
		Current average physical activity	
			More than three times per week and >30 minutes per week
			Less than three times per week and <30 minutes per week
**Diagnosis**			
		Normal weight with no history of being overweight	
		Normal weight with history of being overweight	
		Overweight with no intention to lose weight	
		Overweight due to improper diet with intention to lose weight	
		Overweight due to improper diet and lack of physical activity with intention to lose weight	
		Overweight due to lack of physical activity with intention to lose weight	
		Overweight due to improper self-monitoring with intention to lose weight	
		Obese due to improper diet and lack of physical activity	
		Obese due to improper diet	
		Obese due to lack of physical activity	

**Outcome identification**		
	Target weight	
		Proper target weight
		Improper target weight
	Durationof weight loss	
		Proper duration of weight loss
		Improper duration of weight loss
**Implementation**		
	Initial implementation	
		Educate about weight management
		Educate about low-calorie diet
		Educate about low-calorie diet and physical activity
		Educate about physical activity
		Educate about maintaining weight
	Intermediate implementation	
		Advice about low-calorie diet
		Advice about low-calorie diet and physical activity
		Advice about physical activity
		Advice about self-monitoring
		Transfer to obesity specialist
		Recommend the proper rate of weight loss and normal weight range
**Evaluation**		
	Reason for failure to lose weight	
		Diet nonadherence
		Physical-activity nonadherence
		Self-monitoring nonadherence
		Unknown reason
	Final evaluation	
		Successful weight control
		Unsuccessful weight control
